# Smoldering in the sanctuary: HIV-associated brain injury in the ART era

**DOI:** 10.1172/JCI204831

**Published:** 2026-05-01

**Authors:** Paraskevas Filippidis, Shelli F. Farhadian

**Affiliations:** 1 Department of Pathology, Yale School of Medicine, New Haven, Connecticut, USA.; 2 Infectious Diseases Service, Department of Medicine, Lausanne University Hospital and University of Lausanne, Lausanne, Switzerland,; 3 Department of Internal Medicine, Section of Infectious Diseases, and; 4 Department of Neurology, Yale School of Medicine, New Haven, Connecticut, USA.

## Abstract

Although combination antiretroviral therapy (ART) has dramatically reduced the incidence of severe HIV-associated neurological disease, the central nervous system (CNS) remains a viral sanctuary in which inflammation and brain injury persist despite systemic viral suppression. Here, we synthesize evidence that ongoing HIV-associated brain injury is sustained not primarily by unchecked viral replication but by persistent viral transcription from defective proviruses, immune-mediated synaptic dysfunction driven by bystander activation, and long-lived microglial reprogramming shaped by epigenetic “training.” We highlight how emerging single-cell multiomics and “liquid biopsy” approaches are redefining our understanding of the CNS reservoir at high resolution. We further discuss the growing emphasis on biologically anchored, molecularly defined disease subtypes as a means to disentangle HIV-specific pathology from the confounding overlap of aging and multimorbidity, which have increased in the ART era. Finally, we underscore the necessity of human-centered translational studies to validate preclinical findings, outlining how these molecular insights pave the way for precision therapeutics and CNS-targeted cure strategies.

## Introduction

Neurologic complications were among the earliest and most devastating manifestations of HIV infection, recognized within the first years of the epidemic as sentinel features of advanced disease (1, 2). In the era before antiretroviral therapy (ART), the burden of central nervous system (CNS) involvement was striking. HIV-associated dementia developed in approximately 15%–20% of individuals with advanced AIDS, and milder neurocognitive syndromes were observed in up to 30%–50% of patients with uncontrolled viremia (3, 4). Neurologic decline often paralleled immunologic collapse, and the presence of dementia portended shortened survival. These observations firmly established the brain as a major target of HIV pathogenesis and positioned neuroAIDS as a defining complication of untreated infection (5, 6).

The advent of combination ART dramatically altered this landscape. Effective systemic viral suppression has led to a marked decline in the incidence of HIV-associated dementia and other severe opportunistic neurologic syndromes. In contemporary cohorts, frank HIV dementia is now rare in high-resource settings where early diagnosis and durable viral suppression are common (3). However, the elimination of severe dementia has not equated to restoration of normal brain health. Depending on cohort characteristics and diagnostic criteria, 10%–60% of people with HIV (PWH) continue to meet criteria for some form of neurocognitive impairment (NCI), though the clinical relevance and biological specificity of these classifications remain debated (7–10). Even among virally suppressed individuals who are neurologically asymptomatic, structural neuroimaging abnormalities, cerebrospinal fluid (CSF) immune activation, and molecular evidence of CNS injury persist (11–14). These observations underscore a central paradox of the ART era: while systemic replication is effectively controlled, the CNS remains biologically perturbed.

In parallel, the demographic transformation of the HIV epidemic — with increasing life expectancy — has introduced the intersecting influences of aging, vascular disease, substance use, polypharmacy, and social determinants of health, all of which independently affect brain integrity (15–21). As a result, contemporary HIV-associated CNS disease reflects not only residual viral effects but also the convergence of viral legacy, immune dysregulation, and age-related comorbid processes.

In this Review, we examine HIV neuropathogenesis across the treatment continuum, contrasting the catastrophic inflammatory encephalitis of the pre-ART era with the subtler yet biologically active injury that characterizes treated infection. Rapid advances in single-cell multiomics, spatial transcriptomics, ultrasensitive reservoir assays, and multimodal neuroimaging are transforming our ability to interrogate the CNS reservoir and its downstream consequences in living individuals. Here we synthesize current understanding of how persistent viral transcription from intact and defective proviruses, immune-mediated synaptic dysfunction, and epigenetically reprogrammed microglia sustain CNS injury despite ART. Finally, we discuss evolving conceptual frameworks — including HIV-associated brain injury biotypes — and emerging translational approaches that aim to move the field from descriptive epidemiology toward mechanism-based precision therapeutics.

## HIV’s impact on the CNS before treatment

### CNS viral entry and infection persistence.

HIV enters the CNS within days to weeks of systemic infection (22). The predominant mechanism of HIV neuroinvasion likely involves the transendothelial migration of infected immune cells, a process augmented by HIV gp120’s deleterious effects on tight junctions of the blood-brain barrier (23). Early studies, using indirect methods such as in vitro assays and peripheral blood immunophenotyping, suggested that monocytes — particularly CD14^+^CD16^+^ subsets — were the primary immune cell type carrying HIV to the CNS during early infection (24–26). Likewise, in autopsy studies of both simian immunodeficiency virus–infected primates and individuals with HIV, replicating virus in the CNS is detected primarily in perivascular (monocyte-derived) macrophages and microglia (27, 28), further implicating myeloid cells in establishing and sustaining HIV infection in the CNS.

While monocyte/macrophage trafficking represents the canonical route of HIV neuroinvasion, accumulating evidence indicates that infected CD4^+^ T cells further contribute to the establishment and sustainment of CNS infection (as reviewed in ref. 29). Phylogenetic analyses of paired CSF and plasma viral sequences demonstrate that early CNS viral populations require cells with high CD4 density for cell entry (a density found on T cells but not myeloid lineage cells) and that these sequences are genetically similar to T cell–tropic strains (30). Viral strains capable of infecting T cells but not monocytes precede the emergence of strains capable of infecting cells with low CD4 density, such as macrophages, later in infection, and support a model in which infected T cells establish the first wave of CNS infection (31). Macrophages may also become infected through the capture and engulfment of HIV-infected CD4^+^ T cells (32). Evidence from human postmortem and CSF studies further shows that HIV DNA is detectable in CNS-infiltrating T cells before treatment, underscoring a critical role of T cells in founding the CNS reservoir (33, 34).

Once HIV is established in the CNS, it is sustained through localized replication within the CNS, in addition to ongoing trafficking of infected cell–associated and cell-free virus from the periphery. Phylogenetic analyses performed on specimens obtained from viremic individuals demonstrate that HIV sequences isolated from CSF often diverge from contemporaneous plasma sequences, providing evidence of localized viral replication and adaptation within the CNS (31).

Collectively, these findings indicate that infected T cells play a key role in the earliest stages of HIV neuroinvasion, seeding infection in the brain that is later maintained and amplified by macrophages and microglia. This knowledge has informed the basis for current studies that focus on macrophages and microglia as the primary source of residual viral RNA and protein production in PWH who are on suppressive ART.

### Neuropathological impact of HIV in the brain.

Autopsy studies from the pre-ART era provide the clearest insights into how sustained, uncontrolled HIV replication damages the CNS. Brain tissue from many individuals who died with untreated HIV infection exhibit a characteristic constellation of pathological findings collectively termed HIV encephalitis (HIVE). HIVE is defined by microglial nodules containing HIV antigens, astroglial activation, myelin loss, and brain atrophy (1, 35). These lesions are most prominent in deep gray matter and subcortical white matter, and their presence strongly associates with clinical HIV-associated dementia before death (2, 36). Although neurons are rarely infected, autopsy investigations consistently demonstrate loss of synapses and dendritic architecture, as well as axonal injury, supporting the model that neurodegeneration arises largely from inflammatory and virally toxic cascades rather than direct neuronal infection (37). Beyond HIVE, several forms of white-matter pathology were frequently observed in pre-ART specimens, including vacuolar encephalopathy, consisting of intramyelinic vacuoles with relatively intact axons and diffuse leukoencephalopathy (38).

Importantly, many individuals with severe cognitive impairment had no or only minimal discrete lesions visible on routine brain histology (5, 6). When compared with the brains of PWH with NCI and HIVE, these brains show different transcriptional and protein profiles, implying different biological mechanisms underlying similar clinical phenotypes (39, 40).

Phylogenetic studies of virus within distinct brain regions further shows that HIV replication varies markedly across brain regions and evolves independently within the CNS, reinforcing the concept of compartmentalization in natural history infection (41). Thus, early autopsy studies established the CNS as both a key target and reservoir of HIV, with inflammation-driven neuronal and synaptic damage underlying the high burden of cognitive impairment prior to effective therapy.

## HIV in the brain after ART: host immunity

The clinical expression of HIV-associated CNS disease has changed dramatically with the advent of potent ART. Although severe HIV-associated CNS diseases such as HIV dementia are now rare (42), a substantial subset of PWH continue to exhibit persistent CNS immune activation, microglial dysregulation, and cognitive or neuropsychiatric symptoms despite long-term viral suppression. Modern work — including structural neuroimaging, CSF biomarker profiling, and single-cell immunologic studies — has shifted the focus from uncontrolled viral replication to the mechanisms by which HIV alters CNS biology despite ART.

### CSF biomarkers of residual neuroinflammation and injury.

Despite durable viral suppression with ART, persistent CNS immune activation remains a hallmark of HIV neuropathogenesis and is consistently linked to NCI. Much of the evidence for ongoing neuroimmune dysfunction derives from CSF studies. It is important to note that CSF is not a direct surrogate of brain parenchyma, but rather acts as a dynamic interface enriched for immune surveillance, trafficking cells, and soluble mediators that integrate signals from multiple CNS compartments. These CSF studies of PWH on ART demonstrate sustained elevations in monocyte/macrophage activation markers (e.g., neopterin, soluble CD14, soluble CD163) and inflammatory cytokines and chemokines (e.g., IFN-γ, IL-1α, CCL2) (11), often paired with markers of glial activation, neuronal injury, and neurodegeneration such as neurofilament light chain, YKL-40, TREM2, and glial fibrillary acidic protein (12, 13). Systematic reviews and targeted studies identify a core set of CSF analytes — including CCL2/monocyte chemoattractant protein-1, IL-8/CXCL8, and CXCL10/IP-10 — associated with NCI, though effect sizes and directions vary, reflecting biological heterogeneity and limitations of clinical phenotyping (11, 43). High-throughput proteomic approaches have further reinforced this framework, revealing broad signatures of residual CNS immune activation and axonal injury that persist despite ART (44). Ultrasensitive platforms such as single-molecule array assays now enable detection of low-abundance CNS injury biomarkers in blood and CSF and may play an important role in future longitudinal and interventional studies. However, soluble biomarkers alone provide an indirect and sometimes inconsistent view of disease mechanisms, underscoring the need to integrate these findings with insights from cellular analyses to more precisely define the immune drivers of ongoing CNS injury in treated HIV.

### Persistent microglial activation despite ART.

Within the CNS, two principal macrophage populations drive HIV pathogenesis: yolk-sac–derived parenchymal microglia, which self-renew locally, and border-associated macrophages (perivascular, meningeal, and choroid plexus), which are periodically replenished by circulating monocytes (45–47). Together, these CNS myeloid populations serve as central cellular targets and long-lived reservoirs for HIV.

**Figure 1:**
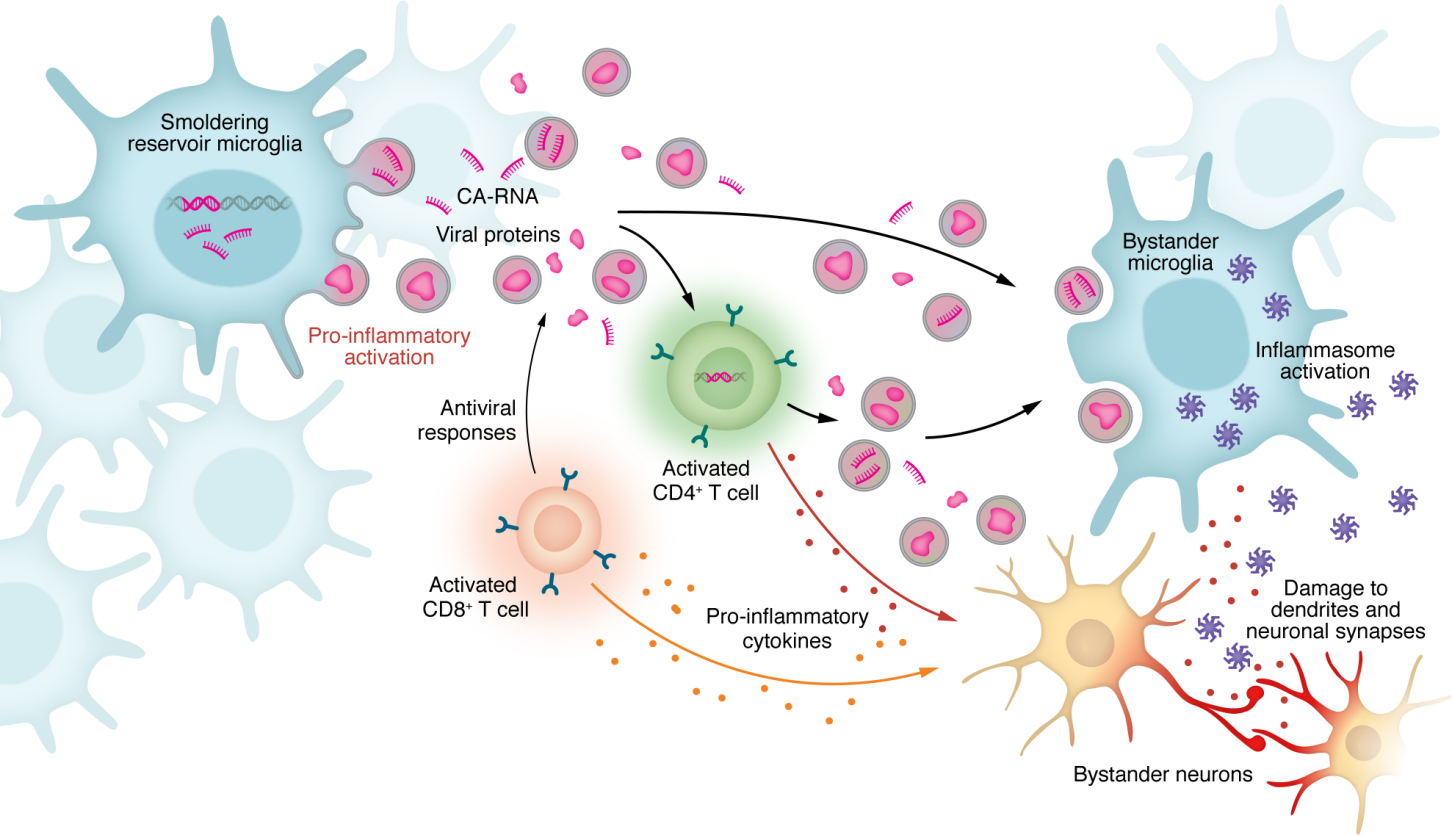
The “smoldering secretome”: mechanisms of bystander injury during viral suppression. Even in the absence of productive viral replication, HIV reservoirs and persistent immune activation in the CNS drive ongoing neurotoxicity. The “smoldering” reservoir microglia (left) harbors defective or silenced proviruses that retain the capacity to transcribe viral RNA and produce viral proteins (such as Tat, Nef, or gp120) without assembling infectious virions. These viral products, along with cell-associated RNA (CA-RNA), are released into the extracellular space as soluble molecules or packaged into EVs. Adaptive immune cells also contribute to this compartmentalized inflammation. Activated CD4^+^ T cells, which may also harbor HIV DNA, interact with microglia and may release both pro-inflammatory cytokines and viral transcripts or proteins. Simultaneously, activated CD8^+^ T cells may play a role both in viral clearance and in fueling inflammation by secreting soluble inflammatory factors, such as IFN-γ. This “viral secretome” traffics to uninfected bystander cells, including microglia and neurons. Uptake of these toxic EVs triggers inflammasome activation and cytokine release in bystander microglia and induces synaptic damage, sustaining neuroinflammation and brain injury despite ART.

Even under suppressive ART, these cells sustain neuropathogenesis through both direct infection and indirect “bystander” activation. While infected microglia (estimated to comprise less than 0.5% of the total population) (48) specifically upregulate S100 family genes (which modulate inflammatory responses in the CNS) (49, 50), and various chemokines and IFN-related pathways, uninfected bystander cells also adopt inflammatory phenotypes, responding to local inflammatory cues, such as viral proteins, and leading to synaptodendritic injury (48, 50) (Figure 1). This is further corroborated by studies using translocator protein (TSPO) PET imaging. TSPO is expressed on activated glia; increased TSPO radiotracer binding is consistently found in PWH on suppressive ART compared with controls, even in asymptomatic individuals (51, 52). It should be noted, though, that the cellular specificity of TSPO in human studies is limited, as TSPO is also expressed by astrocytes and endothelial cells. This underscores the need to interpret TSPO signal as a composite marker of neuroinflammation and highlights the importance of developing next-generation tracers capable of distinguishing microglial from astroglial activation in vivo.

Recent single-nucleus RNA-seq (snRNA-seq) and ATAC-seq studies demonstrate that chronic HIV drives 3D chromatin reconfiguration and promotes pro-inflammatory, neurodegeneration-related, and senescent gene programs even in the absence of overt viral replication (48, 53). A key driver of this primed state is the NLRP3 inflammasome. Viral proteins Tat, gp120, and Vpr prime and activate the inflammasome, promoting caspase-1 cleavage and release of IL-1β and IL-18 (54–58). These processes recruit additional immune cells and propagate inflammatory signaling to bystander neurons and glia, further amplifying neurodegeneration. Mechanistically, this persistence of abnormal microglial proliferation and activation may be driven by trained immunity — a process of long-term functional reprogramming of innate immune cells via epigenetic remodeling (59); however, further studies are required to understand the role of epigenetic alterations in HIV-associated microglial activation.

Beyond bona fide resident brain microglia, monocyte-derived microglia-like cells are present in the adult human brain and in CSF and may further contribute to HIV neuropathogenesis (60). Recent CSF profiling studies have identified CD204 as a marker to identify and isolate these cells from the CSF of PWH (14, 61). Thus, CD204-expressing cells in CSF may provide a window into microglial activation states in vivo and represent a promising candidate biomarker for HIV-associated brain injury (14, 61). However, further immunophenotyping is required to define their precise role in HIV pathology.

### Cell-mediated immunity.

HIV is fundamentally T cell tropic, but CNS impairment extends beyond the direct effects of CD4^+^ T cell depletion. Even in the setting of durable virologic suppression, trafficking, activation, and functional skewing of lymphocytes within the CNS sustain compartmentalized neuroinflammation (14, 62, 63). Critically, because brain tissue is rarely sampled, our understanding of these dynamics relies heavily on the study of CSF-derived cells.

In the CNS, CD8^+^ T cells play a dual role. While HIV-specific CD8^+^ T cells — including highly activated and MHC class I–restricted CD4^dim^CD8^bright^ subsets — limit viral replication and correlate with reduced inflammation (64), generalized CD8^+^ T cell activation drives pathogenesis. Longitudinal and single-cell RNA-seq (scRNA-seq) analyses show that activated CD8^+^ T cell phenotypes persist in CSF despite treatment (14, 63, 65). This chronic activation, alongside CD4/CD8 imbalance and increased IFN-γ production, correlates with cognitive impairment and white-matter abnormalities on MRI (62, 66). A similar process is observed in peripheral blood, where T cell senescence and exhaustion associate with both cognitive impairment and brain atrophy (67, 68).

### Humoral immunity.

B cells are increasingly recognized as active participants in shaping CNS inflammation in ART-treated PWH. In the periphery, deficits in resting memory B cells and impaired antigen-specific responses are present despite ART (69, 70). Likewise in the CSF, despite systemic viral suppression, the B cell chemoattractant CXCL13 is frequently elevated and associates with inflammatory biomarkers and cognitive impairment (71). Similarly, intrathecal IgG synthesis is elevated and correlates with both blood-CSF barrier dysfunction and NCI (72, 73), suggesting persistent intrathecal B cell activation and antibody production as potential drivers of CNS inflammation, independent of plasma HIV replication.

High-resolution antibody profiling further shows that CSF antibodies are distinct from those in the periphery — enriched for HIV-specific reactivity but functionally impaired — and are resistant to ART-induced changes (74). Not all dysregulated antibody responses are HIV specific, though: intrathecal anti-EBV IgG and autoantibodies (e.g., anti-CD4) in the CSF correlate with markers of neuroinflammation, neuronal injury, and clinical symptoms (75–77). Collectively, these findings support a model in which persistent B cell activation, intrathecal antibody production, and immune complexes may interact with activated T cells and microglia — via antigen presentation, Fc receptor engagement, and chemokine signaling — to sustain compartmentalized neuroinflammation in the ART era. Further studies characterizing B cell phenotypes and functions beyond antibody production remain essential to refine this model and define new therapeutic targets.

### Nonimmune mechanisms of HIV-associated brain injury.

Immunologic host factors represent a major focus in HIV neuropathogenesis; however, these processes occur within a broader landscape of interacting nonimmune mechanisms that also contribute to brain vulnerability. Emerging evidence highlights roles for mTOR pathway dysregulation, which affects autophagy, apoptosis, and neuroimmune interactions (78, 79); Alzheimer’s disease–like pathogenic processes, including alterations in amyloid and tau pathways that may be accentuated in aging PWH (80–82); mitochondrial dysfunction, impairing neuronal bioenergetics and promoting oxidative stress (83–85); and gut microbiome perturbations, which fuel systemic immune activation and altered neurotransmitter signaling (86–88). These mechanisms provide complementary frameworks for understanding the multifactorial nature of HIV-associated brain injury; however, a detailed discussion of them is beyond the scope of the present Review.

## HIV in the brain after ART: viral products

In the ART era, HIV neuropathogenesis has shifted from acute viral cytopathicity to reservoir-driven neuroimmune dysregulation. Persistent HIV proviral DNA reservoirs within the CNS sustain low-level expression of viral transcripts and proteins despite ART. These residual viral products exert direct neurotoxic effects and chronically engage innate and adaptive immune pathways, resulting in bystander neuronal injury, synaptic dysfunction, and ultimately cognitive impairment (89–91). Indeed, HIV-specific CD8^+^ T cells remain in the CNS even after years of durable ART (64, 92), suggesting viral protein expression even in the presence of ART and clinically suppressed viral load.

### Proviruses in the CNS during ART.

The characterization of the HIV-1 reservoir within the CNS has transitioned from simple DNA quantification to sophisticated assays capable of distinguishing between intact and defective proviruses. Current methodologies primarily utilize the Intact Proviral DNA Assay (IPDA) and near-full-length genome sequencing (nFGS) to map this landscape (93, 94). The IPDA employs droplet digital PCR with multiplexed probes targeting the highly conserved packaging signal (Ψ) and envelope (env) regions; proviruses are classified as “intact” when both targets are detected, while those missing one or both are identified as defective (93). For definitive characterization, nFGS is employed as the gold standard, involving limiting dilution of DNA to the single-genome level followed by long-range PCR and deep sequencing to identify lethal defects like internal stop codons and hypermutations (94, 95). Recent studies have successfully applied these techniques to both CSF and postmortem brain tissue, revealing that while the majority of CNS proviruses are defective, a stable population of genome-intact, potentially replication-competent virus persists in microglia and other resident cells despite long-term ART (96, 97). Although overall proviral burden in brain tissue is low compared with lymphoid compartments (98), intact genomes are reproducibly detected across multiple brain regions, including frontal white matter and cortex, and in CSF, where approximately 1.7% of total proviral DNA is intact, irrespective of ART status (96, 99, 100). Critically, CNS proviral burden correlates with cognitive deficit scores, implicating these persistent infected cells in ongoing NCI (101–103).

Most HIV provirus in the brain has been localized to microglia and other myeloid lineage cells (96, 100, 104, 105), with approximately 0.5% of these cells harboring HIV DNA, including inducible replication-competent virus (48, 102, 105, 106). Viral subtype may also influence reservoir burden, with subtype C–infected brains showing lower intact proviral frequencies compared with subtype B–infected brains (107). In addition to resident myeloid cells, a distinct population of CD4^dim^CD8^bright^ T cells has been shown, in an animal model, to traffic to the brain and harbor replication-competent HIV, suggesting a role for lymphocyte-mediated HIV neuroinvasion and CNS persistence alongside established myeloid reservoirs (108).

The vast majority of cells in the brain are glial cells, predominantly astrocytes. Thus, there has been considerable attention paid to whether these non-CD4-expressing cells are infected by HIV and whether they serve as a nonimmune CNS reservoir. The results are conflicting. Although a seminal RNAscope and DNAscope study of postmortem brains did not detect HIV in any brain astrocytes (102), studies using more sensitive assays have consistently detected integrated HIV DNA in a subset of astrocytes, even among PWH on long-term suppressive ART (97, 109–112). Astrocytes appear to be infected via receptor-mediated endocytosis and by cell-to-cell contact from infected lymphocytes (113). The consequences of the astrocyte reservoir are potentially important: animal studies suggest that astrocytes are capable of producing virions that are trafficked to the periphery via infected CD4^+^ T cells and may contribute to viral reseeding outside of the CNS (110).

### Persistent HIV transcription in the CNS.

Although ART blocks productive HIV replication, viral transcription persists in the CNS of virally suppressed PWH. Defective proviruses, long assumed to be silent, dominate brain-resident HIV genomes and retain transcriptional competence, ultimately enabling the production of aberrant or truncated proteins with inflammatory potential (96, 100, 114–117).

In accord with studies of HIV DNA, postmortem studies consistently identify microglia as the dominant source of HIV RNA in the brain (48, 106). These HIV RNA–positive microglia may exhibit a distinct inflammatory phenotype, despite broadly similar chromatin accessibility across microglial subsets; however, larger studies with greater numbers of HIV RNA–positive cells are required to draw more definitive conclusions (48). In frontal cortex tissue from virally suppressed and nonsuppressed individuals, low but detectable HIV transcripts were detected in most ART-treated PWH, in the range of tens to hundreds of copies per 10^6^ cells, with evidence of transcriptional completion in a subset of suppressed individuals (118). Notably, HIV p24^+^CD68^+^ myeloid cells were identified in suppressed brains, indicating that viral protein production persists in at least some individuals despite ART. Although a proximal elongation block was observed — more pronounced in virally suppressed participants — these findings suggest that CNS proviruses are transcriptionally restricted rather than fully silenced, reinforcing the concept of a “smoldering” reservoir in the brain capable of sustaining chronic immune activation (118).

In the CSF, HIV RNA is found primarily in central memory CD4^+^ T cells (14, 119, 120). This discrepancy — with brain studies showing HIV RNA in myeloid cells and CSF showing most HIV transcription in T cells — likely reflects the overall immune cell composition of CSF compared with the brain, with CSF consisting almost entirely (~90%) of T cells (14, 61), compared with very few infiltrating and brain-resident T cells (121). A subset of HIV RNA–containing T cells in the CSF are members of T cell clonal populations present in both blood and CSF (120). The presence of HIV RNA–producing cells sharing TCR sequences across blood and CSF compartments even after decades of ART lends further support to the notion that infected T cells traffic between tissue compartments and that maintenance and expansion of infected T cell clones contribute to the maintenance of the CNS reservoir in PWH on ART.

Beyond cellular transcripts, extracellular vesicles (EVs) in CSF contain multiple classes of HIV RNAs, including readthrough, TAR, long-LTR, Pol, Nef, and Tat–Rev, at higher levels than in blood. These EV-associated transcripts correlate strongly with global and domain-specific cognitive impairment (122).

### The synaptoxicity of HIV.

EVs containing viral proteins such as Tat, Nef, or gp120 persist in the CNS despite ART (97, 123, 124). In animal models, sustained CNS exposure to gp120 or Tat leads to cognitive impairment (125–128). Henderson et al. detected Tat in the CSF of approximately one-third of ART-treated individuals, packaged within EVs with preserved transactivation activity. Importantly, Tat levels did not correlate with CSF viral load in individuals with transient CSF HIV escape, suggesting that EV-associated Tat may originate from viral reservoirs and be released independently of detectable viral replication (124). Nef, which is also released in EVs from HIV-infected immune and glial cells, has similarly been implicated in CNS injury (129). Although direct infection of microglia can trigger focal release of soluble neurotoxic viral proteins, the widespread synaptic damage observed during ART suppression is likely in large part sustained by EV-mediated transfer of viral proteins to uninfected cells (130–132).

Residual viral proteins drive synaptodendritic injury through multiple, non–mutually exclusive mechanisms. Gp120 and Tat alter NMDA receptor signaling, disrupting calcium homeostasis and promoting neuronal toxicity (133–135). Inflammatory cytokines released from activated bystander microglia, including TNF-α, further exacerbate NMDA receptor dysregulation activity on neurons (136). HIV proteins may also impact the structural stability of dendritic spines by altering host proteins such as the E3 ubiquitin ligase Mdm2 (133, 137). In addition, exposure of uninfected astrocytes to viral proteins impairs glutamate transport, contributing to synaptic loss (138–140).

Together, these findings support a model in which the CNS reservoir functions as a chronic “toxic hub,” where a relatively small population of infected cells drives widespread synaptodegeneration through pathological activation of surrounding uninfected cells. Older ART regimens may also induce synaptic loss (141). However, studies directly assessing synaptic integrity in PWH on suppressive ART remain limited, and much of our understanding of viral protein–mediated synaptic toxicity is derived from animal and in vitro models. Emerging neuroimaging approaches, such as PET imaging with the synaptic vesicle glycoprotein 2A (SV2A) tracer, enable in vivo measurement of synaptic density and may be informative to tracking synaptodendritic injury over the course of HIV and its treatment (142).

## Clinical challenges and frameworks: HIV, aging, and comorbidities

### Evolving concepts: from HAND to HIV-associated brain injury.

The historical framework of HIV-associated neurocognitive disorders (HANDs) was developed in the pre-ART era and has limited specificity today. HAND classifications often label 20%–60% of PWH as cognitively impaired, largely because the criteria include asymptomatic neurocognitive impairment, which may lack clinical relevance (3, 4). These prevalence estimates do not align with the clinical experience of providers and likely overestimate clinically meaningful disease in the era of viral suppression.

**Figure 2:**
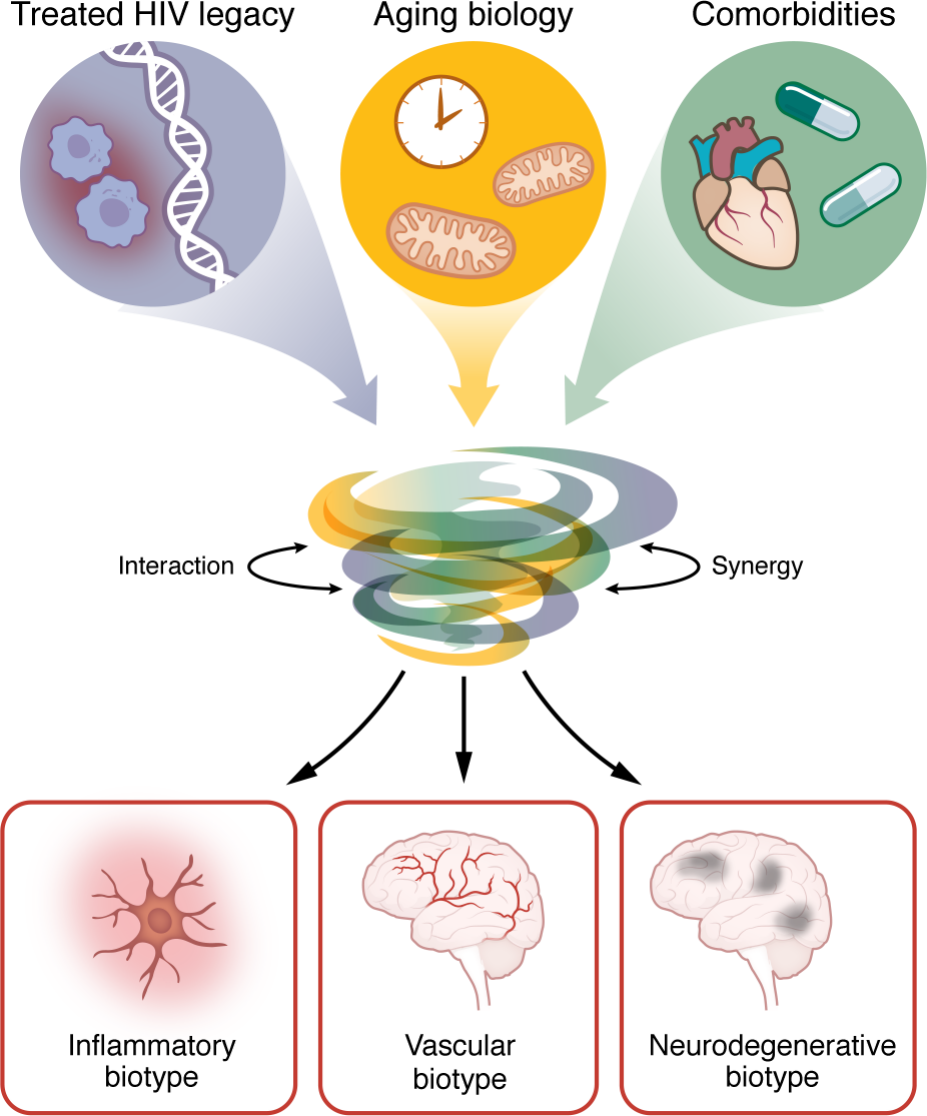
Evolving frameworks: the convergence of HIV, aging, and comorbidities into distinct biotypes. Moving beyond the historical HAND classification, modern HIV-associated brain injury is conceptualized as a heterogeneous condition driven by the convergence of three distinct upstream drivers: the legacy of treated HIV (including reservoir persistence, residual inflammation, neuronal injury, and glial activation), the biology of aging (mitochondrial dysfunction, senescence), and the burden of non-AIDS comorbidities (cardiovascular disease, polypharmacy, substance use, coinfections, and social factors). These drivers do not act in isolation but interact synergistically to alter CNS homeostasis. This convergence resolves into phenotypic profiles, or biotypes. Recognizing these complex interactions and biological profiles is essential for identifying risk factors and opportunities for targeted interventions and precision therapeutics.

Given the mismatch between HAND categories and modern biology, there is a shift toward the construct of HIV-associated brain injury, which prioritizes objective evidence of CNS pathology — such as neuroimaging abnormalities, persistent immune activation, and microglial signatures — over neuropsychological thresholds alone (143). This biologically anchored approach aims to categorize PWH into distinct biotypes based on their underlying drivers of injury, such as inflammatory, vascular, or neurodegenerative dominant profiles, acknowledging the multifactorial interplay between HIV legacy effects and non-HIV comorbidities (113–115) (Figure 2).

### The aging brain: comorbidities and inflammatory drivers.

With increased life expectancy, aging and frailty have emerged as dominant determinants of brain health in PWH. Virally suppressed PWH often exhibit premature or accelerated aging, with higher prevalence and earlier onset of age-associated conditions (15–18, 144–147). Population-level studies using electronic medical record–based diagnoses reveal both a higher prevalence and an earlier age of onset for age-associated dementias in people with HIV, even in the ART era (147, 148). Structural MRI studies support this model, revealing accelerated cortical and white-matter abnormalities in older PWH, particularly among those with high comorbidity burdens (149).

PWH exhibit higher rates of ischemic stroke and cerebral small-vessel disease — including white-matter hyperintensities, lacunar infarcts, and microbleeds — even under viral suppression (150, 151). Mechanistically, chronic inflammation and endothelial dysfunction fuel this damage and delineate a specific neurocognitive profile defined by executive dysfunction and slowed processing speed (152–155).

Stimulants (methamphetamine, cocaine), opioids, and alcohol remain potent amplifiers of host-mediated CNS injury. These substances disrupt blood-brain barrier integrity, enhance microglia driven neuroinflammation, and facilitate the transmigration of HIV-infected monocytes into the CNS (156–158). Chronic coinfections (EBV, CMV, hepatitis C virus) further modulate CNS inflammation (159–162). Sleep disturbance, which is nearly ubiquitous in chronic HIV, may create a feed-forward loop: disrupted sleep impairs glymphatic clearance and alters microglial reactivity, which in turn heightens neuroinflammation and worsens depressive symptoms and cognitive impairment (163–165).

Crucially, the preservation of brain health in PWH is complicated by the cumulative iatrogenic burden of polypharmacy. As individuals age with HIV, the number of prescribed medications increases substantially, often exceeding that of HIV-negative peers, and polypharmacy has been independently associated with frailty, slowed gait, falls, and impaired cognition (19, 20). These associations persist even after accounting for comorbidity burden, suggesting direct medication-related neurocognitive effects. Beyond the known neuropsychiatric effects of certain ART agents like efavirenz, the high burden of non-HIV medications (e.g., benzodiazepines, sedative-hypnotics) — particularly those with anticholinergic properties — is linked to accelerated cognitive decline and increased dementia risk (166–169). These factors underscore the need for a brain health–centered approach to aging PWH, prioritizing deprescribing, minimizing CNS-active agents, and integrating geriatric care (170, 171).

### Novel therapeutic approaches toward neuroprotection and HIV cure.

Clinical trials of minocycline (172, 173), selegiline (174), and tesamorelin (175), and maraviroc intensification (176), have been unsuccessful so far. The limited success of these prior, primarily antiinflammatory focused trials likely reflects intervention at a late disease stage, absence of molecular stratification, and targeting of downstream inflammation rather than upstream drivers such as persistent viral transcription and glial reprogramming. Modern efforts toward CNS-targeted cure increasingly emphasize combinatorial approaches that reduce reservoir burden (177). Latency-reversing agents remain foundational to “shock-and-kill” strategies (178), but CNS implementation requires caution given distinct reservoir cell types and risks of catastrophic inflammatory neurotoxicity (177, 179). Broadly neutralizing antibodies (bNAbs) provide antiviral and immunomodulatory activity but have limited CNS penetration (180, 181). Gene editing and cell-based approaches remain promising but face challenges in delivery across the blood-brain barrier, off-target effects, and targeting long-lived myeloid reservoirs (181, 182). Emerging, mechanism-based therapies include nanoparticle-based delivery of ART, bNAbs, antioxidants, and antiinflammatory agents (182–185); small-molecule inhibitors of myeloid HIV transcription (186); and CCR2/CCR5 dual inhibitors that target monocyte activation and neuroinvasion (187, 188).

Given that HIV-related brain injury is fundamentally characterized by synaptodendritic injury, the emergence of synaptic regenerative therapies represents a potentially promising therapeutic shift. Pharmaceutical agents that are designed to promote dendritic spine resprouting may help restore synaptic connectivity lost through chronic exposure to viral proteins (189). Similarly, the p75 neurotrophin receptor ligand LM11A-31 has demonstrated preclinical efficacy in HIV models by stabilizing neuronal calcium homeostasis and counteracting pro-apoptotic signaling pathways that contribute to neuropathogenesis (190, 191). By focusing on structural synaptic repair rather than solely on viral suppression, these approaches offer a potential strategy for actively restoring cognitive function, particularly in the aging population of people living with HIV.

## Looking ahead

While modern ART effectively halts systemic replication, the CNS remains a “smoldering” sanctuary where both intact and defective proviruses drive localized immune activation and microglial dysregulation. As the population of PWH ages, these viral legacy effects intersect with cardiovascular disease, metabolic syndrome, and the cumulative burdens of multimorbidity and polypharmacy. To untangle this multifactorial injury, the field must transition from descriptive neuropsychological thresholds toward a biologically anchored framework that identifies distinct biotypes of injury, paving the road to precision therapeutics. The National Institute of Mental Health Biotypes of CNS Complications formalizes this transition by integrating host and viral genomics, high-field neuroimaging, and deep clinical phenotyping, to identify reproducible biological categories (192). Within this framework, advanced neuroimaging and modern approaches for CSF and neuropathologic analyses are crucial.

### Advanced neuroimaging.

High-resolution MRI, PET, and emerging imaging tracers have become instrumental to detecting subtle HIV-associated brain abnormalities in ART-treated PWH. Structural and functional MRI identify gray-matter loss, white-matter abnormalities, and disrupted network connectivity, while newer modalities, such as diffusion MRI, quantitative MRI and MR spectroscopy, probe microstructure, inflammation, and glial alterations, particularly in subcortical regions, such as the basal ganglia and corpus callosum (193–196). Functional and perfusion MRI reveal disrupted resting-state connectivity, altered task-related activation, and abnormal cerebral blood flow (197–199). Small-scale, preliminary PET studies using SV2A ligands demonstrate reduced synaptic density correlating with cognitive performance (200), though these findings require validation in larger, well-powered cohorts. Additionally, TSPO tracers localize increased microglial and astrocytic activation (52), while amyloid and tau ligands reveal pathological deposition in the brains of virally suppressed PWH (201). Magnetoencephalography further uncovers aberrant neural oscillatory dynamics (202). Integrated multimodal imaging and machine learning now aim to generate individualized prediction models of NCI (195, 203), representing a transition from descriptive to mechanistic imaging biomarkers.

*CSF as a “liquid biopsy.”* CSF serves as a vital “window” into the brain. Whereas prior CSF studies primarily focused on the measurement of soluble inflammatory proteins, in an unsuccessful search for a soluble biomarker of HAND, modern studies capitalizing on technical advances in single-cell profiling have begun to reveal the landscape of host immunity in the CNS of PWH through a focus on cells, rather than just soluble proteins, of the CSF. The majority of CSF immune cells, primarily T cells, migrate from the peripheral blood across the blood-CSF barrier at the choroid plexus to provide continuous immune surveillance of the central nervous system. Beyond the systemic circulation, recent research identifies cranial bone marrow as a vital local reservoir that supplies immune cells — particularly myeloid cells and B cells — directly to the meninges and CSF through microscopic vascular channels (204). The meningeal lymphatic system also serves as a specialized conduit that allows for the trafficking and potential recirculation of immune cells between the subarachnoid space and the peripheral lymph nodes (205). However, these alternative sources of CSF cells have not been well studied in the context of HIV infection.

Multimodal platforms integrating transcriptomics, surface proteomics, chromatin accessibility, and histone modification now enable CSF immune cell mapping with a granularity approaching that of brain tissue profiling, allowing for real-time, longitudinal sampling of CSF cells in living people. Coupling these approaches with adaptive immune repertoire sequencing provides high-resolution T cell and B cell receptor clonotypes and insights into clonal architecture, antigen specificity, and trafficking of adaptive immune cells, processes increasingly recognized as central to CNS reservoir persistence (120). scRNA-seq also allows detection of HIV transcripts within CSF-derived cells, supporting models of transcriptionally active but replication-restricted infection driving local immune activation (14, 120), and allowing for the detection of rare, disease-related immune subsets (14, 61).

In addition to single-cell profiling, high-throughput proteomics and metabolomics refine our understanding of neuroinflammation, neuronal injury, and persistent neurometabolic reprogramming across the spectrum of NCI despite viral suppression (11, 44, 206). However, the high dimensionality of these datasets, interindividual variability, and the need for cross-platform harmonization highlight emerging challenges. Future priorities include standardized acquisition and computational harmonization pipelines, larger longitudinal cohorts, and deeper integration with imaging and neuropathology to translate molecular signatures into validated biomarkers and therapeutic targets.

### Neuropathology and postmortem brain tissue studies.

Long-standing autopsy networks such as the National NeuroHIV Tissue Consortium provide deeply phenotyped tissue (207), while rapid-autopsy programs like Last Gift enable sampling within hours of death, preserving RNA, epigenomic architecture, and viable cells for high-resolution virologic and immunologic analyses (208). Modern single-cell multiomics applied to these tissues have refined our understanding of microglial infection in virally suppressed individuals (48), while multiregion atlas efforts further show substantial regional and interindividual heterogeneity in immune activation and HIV-bearing myeloid states, influenced by comorbid factors such as substance use (209). Spatial approaches alongside tissue-level ART drug quantification further define the regional heterogeneity of the reservoir and the variable CNS penetration of ART (102, 210, 211). Looking forward, key priorities include targeted enrichment methods for single-cell HIV RNA and full-length proviral sequencing, high-resolution and 3D reservoir mapping, and cross-species integration of human and animal model data providing opportunities for preclinical testing and rapid translation of CNS-targeted interventions; fresh CSF-brain paired cohorts to track reservoir dynamics across compartments; and incorporation of unbiased pathogen detection to disentangle HIV-specific injury from comorbid CNS pathology (212).

Achieving the precision-medicine goal requires continued emphasis on high-resolution, human-based studies. While animal models offer valuable insights into HIV neuropathogenesis and generate hypotheses, direct interrogation of the human CNS through longitudinal CSF multiomics and rapid-autopsy programs provides the only definitive means to map the viral reservoir and its epigenomic imprinting on resident glia. These human-centered approaches remain essential for validating the unique innate and adaptive immune programs that are central to chronic neuroinflammation and may not be fully recapitulated in animal models (Table 1).

Ultimately, these molecular insights must be viewed through the lens of the global epidemic’s structural realities. Global inequities in neuroHIV diagnosis and management remain substantial. Advanced neuroimaging, CSF biomarker platforms, and neuropathology resources are concentrated in high-income settings, whereas the greatest burden of HIV-associated NCI lies in low- and middle-income countries (213, 214). Early ART initiation and durable suppression clearly mitigate risk of severe HIV-associated NCI, yet disparities in ART access limit these benefits (215). Diagnostic frameworks like the Frascati criteria often misclassify socioeconomic hardships as biological injury (21, 216). Indeed, recent work in South Africa highlights that social determinants of health — including food insecurity, low education, and even structural factors such as lack of indoor bathroom access — exert a major influence on cognitive test performance (21, 216). The finding that social determinants of health can fundamentally dictate cognitive performance serves as a critical warning against the overmedicalization of socioeconomic hardship (213). A rigorous future for neuroHIV research requires a dual commitment: the pursuit of deep, human-derived biological data and the integration of the social context in which these biological processes occur.

## Conflict of interest

The authors have declared that no conflict of interest exists.

## Funding support

This work is the result of NIH funding, in whole or in part, and is subject to the NIH Public Access Policy. Through acceptance of this federal funding, the NIH has been given a right to make the work publicly available in PubMed Central.

- NIH R21MH142338, R01DA060493, R01MH134391.
- Swiss National Science Foundation (grant P500PM_221968 to PF).

## Figures and Tables

**Table 1:**
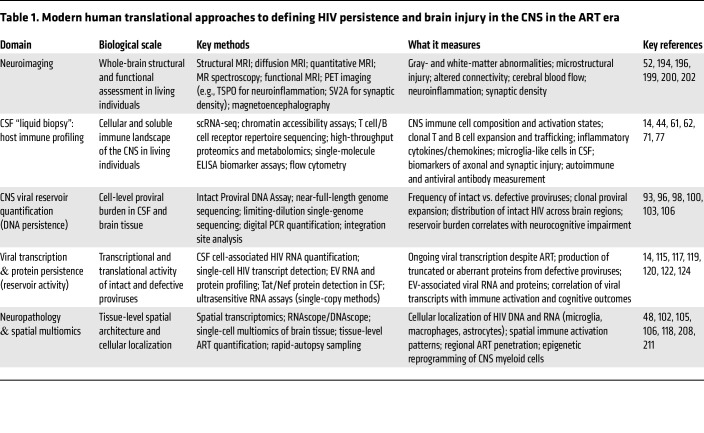
Modern human translational approaches to defining HIV persistence and brain injury in the CNS in the ART era

## Version 1 05/01/2026

Electronic publication
